# Ageing of the Diaphragm Muscle

**DOI:** 10.7759/cureus.6645

**Published:** 2020-01-13

**Authors:** Bruno Bordoni, Bruno Morabito, Marta Simonelli

**Affiliations:** 1 Physical Medicine and Rehabilitation, Foundation Don Carlo Gnocchi, Milan, ITA; 2 Physical Medicine and Rehabilitation, School of Osteopathic Centre for Research and Studies, Milan, ITA; 3 Integrative/Complimentary Medicine, French-Italian School of Osteopathy, Pisa, ITA

**Keywords:** diaphragm, ageing, fascia, skeletal muscle, phrenic nerve, vagus nerve, sarcopenia

## Abstract

The diaphragm muscle is the most important contractile district used for breathing. Like other muscles in the human body, it is subject to ageing and sarcopenia. Sarcopenia can be classified as primary (or age-related) when there are no local or systemic pathologies that cause a functional and morphological detriment of skeletal musculature. Secondary sarcopenia occurs when there is a cause or more pathological causes (illness, malnutrition, immobility) related or unrelated to ageing. In the elderly population, transdiaphragmatic pressure (Pdi) decreases by 20-41%, with a decline in the overall strength of 30% (the strength of the expiratory muscles also decreases). The article discusses the adaptation of the diaphragm muscle to ageing and some other ailments and co-morbidities, such as back pain, emotional alterations, motor incoordination, and cognitive disorders, which are related to breathing.

## Introduction and background

The European Union has the highest proportion of older people (over 65 years of age) compared to other regions [1]. In the US, there are about 48 million inhabitants over 65 years of age. A statistical projection predicts that there will be about 88 million elderly citizens in 2050 in the US [2]. It is believed that the number of elderly people will be higher than the number of children born in the United States by 2030 [3]. In the city of Beijing, China, there are 3.29 million over the age of 65 years (2016 figures) [4]. Ageing is not a problem confined to the most economically developed countries. Malaysia will become a country of people who are predominantly elderly by 2035; In Mexico, the elderly population will have a higher percentage of growth than other Latin American countries by 2050 [5,6]. Tunisia is experiencing an increase in the elderly population and a decrease in overall population growth [7]. India will have a geriatric population of about 198 million people by 2030 [8]. In Egypt, the elderly population will account for about 10.9% of the population by 2026; Nigeria's population of people over 65 years of age will be around 15 million in 2025 [9,10]. The problem is not just the increase in the number of people with advanced age, but also all the physiological adaptations and associated burdens related to it. Sarcopenia happens to be one of the most common among them.

Sarcopenia can be classified as primary (or age-related) when there are no local or systemic pathologies that cause a functional and morphological detriment of skeletal musculature [11]. Secondary sarcopenia occurs when there is a cause or more pathological causes (illness, malnutrition, immobility) related or not to ageing [11]. Healthy elder sarcopenia leads to a decline in muscle coordination, along with decreased strength, speed of contraction, and depletion of muscle mass. This altered contractile function leads to accidental trauma, such as falls. Some 28-35% of healthy elderly people (who are self-employed and without pathologies) experience accidental falling [4]. The resulting traumas account for about 19% of emergency visits; about half of these patients undergo hospitalization; In the US, accidental falls are the third most common cause of non-voluntary traumatic incidents [4]. Fear of falling leads to fear of movement and immobility, which leads to a syndrome called fair-freeze-frailty (FFF). FFF is linked to increased mortality and morbidity, increased falls, and hospitalization [12]. Decreased mobility linked to sarcopenia or FFF is associated with cognitive decline and consequent decline in quality of life and an increased sense of loneliness among the elderly [1,4,13]. In this scenario, we also encounter other recurring conditions: urinary incontinence; dizziness or aged dependent-dizziness; depression and anxiety; and obstructive sleep apnea syndrome (OSAS) [14-17]. Sarcopenia also affects respiratory muscles with functional decrement; there is a decline in maximal inspiratory pressure (MIP) and maximal expiratory pressure (MEP) [18]. The diminished values of MIP and MEP are indicators of sarcopenia. Reduced respiratory function is another parameter for assessing the presence of sarcopenia in healthy older people, and the condition is one of the factors recognized in the definition of the European Working Group on Sarcopenia in Older People (EWGSOP) along with decreased muscle strength, decreased pace speed and loss of muscle [18]. The article reviews the adaptations of the diaphragm muscle with respect to ageing, and it brings to light the altered presence of respiratory function and many symptoms related to ageing. The aim is to cast new light on the importance of the diaphragm in the symptomatic scenario of the elderly and, possibly, to stimulate research in deepening such hypotheses.

## Review

Adaptation of diaphragm muscle in ageing

**Figure 1:**
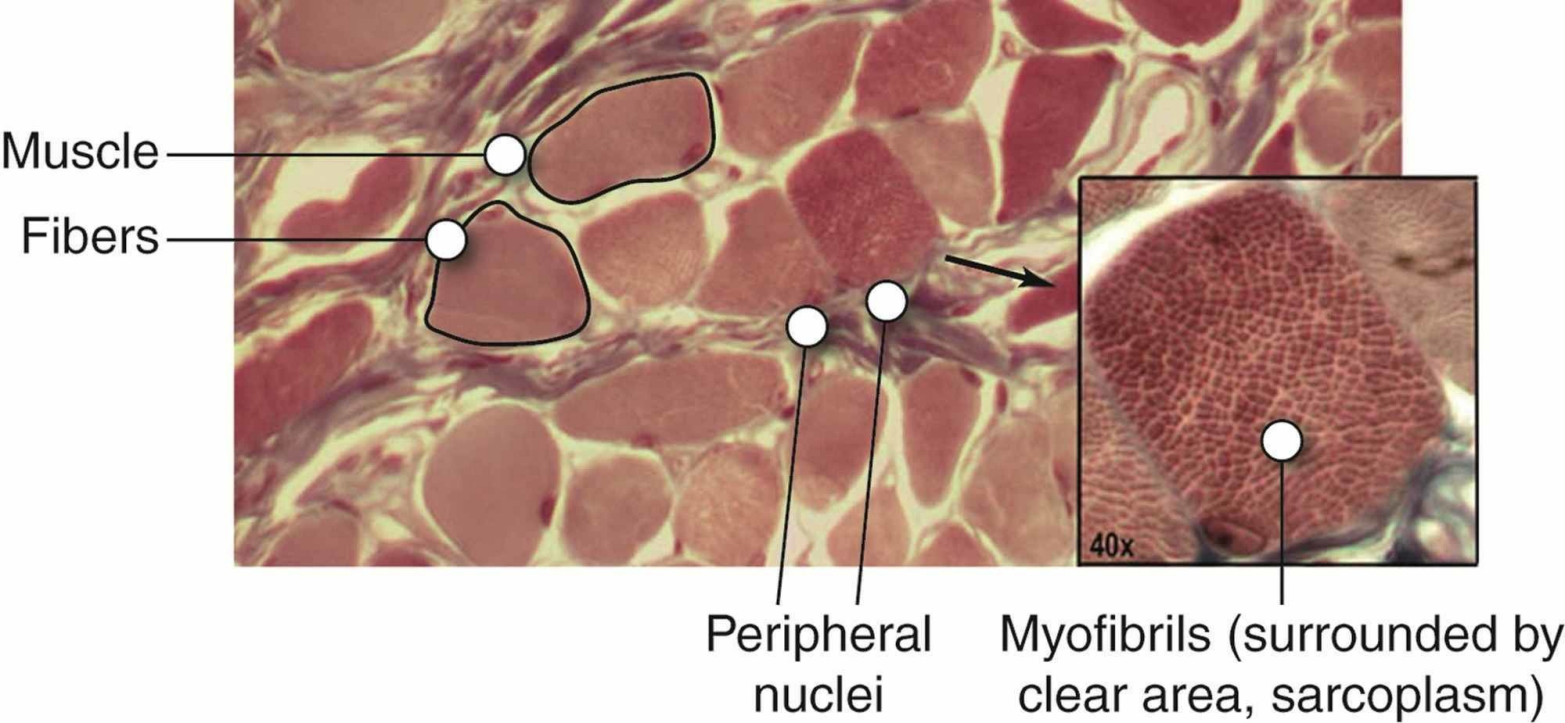
The image of skeletal muscle tissue by electron scanning under a microscope

Ageing has a great impact on posture. It increases the dorsal kyphosis and stiffness of the joints of the chest, and decreases the elasticity of the pulmonary parenchyma; hyperinflation, caused by an increase in residual volume and functional residual capacity, negatively affects the function of the diaphragm muscle [18]. In the elderly population, transdiaphragmatic pressure (Pdi) decreases by 20-41%, with a decline in the overall strength of 30% (the strength of the expiratory muscles also decreases). Sarcopenia is a common condition that combines these adaptations [18]. The aerobic fibers are the ones most saved by sarcopenic processes, while anaerobic fibers are the most negatively affected [19]. Adaptations of different types of fibers are mirrored to the changes in the neurons that make up their motor units. There is a loss of larger spinal neurons or fatigable fast-twitch (of anaerobic motor units or FF) and muscle reinnervation by smaller motor neurons or slow-twitch (ST), with a denervation mechanism and reinnervation [19]. FF is able to generate higher contraction values (peak twitch tension or Pt and maximum tetanic tension or Po), compared to ST [19]. With ageing, the diaphragm muscle becomes less strong; and, for example, the person has difficulty cleaning the upper airways properly with the cough, but his contracting ability for prolonged efforts has a maintained performance. These alterations could be preceded by a decrease in neurotrophic substances (Figure 1).

**Figure 2:**
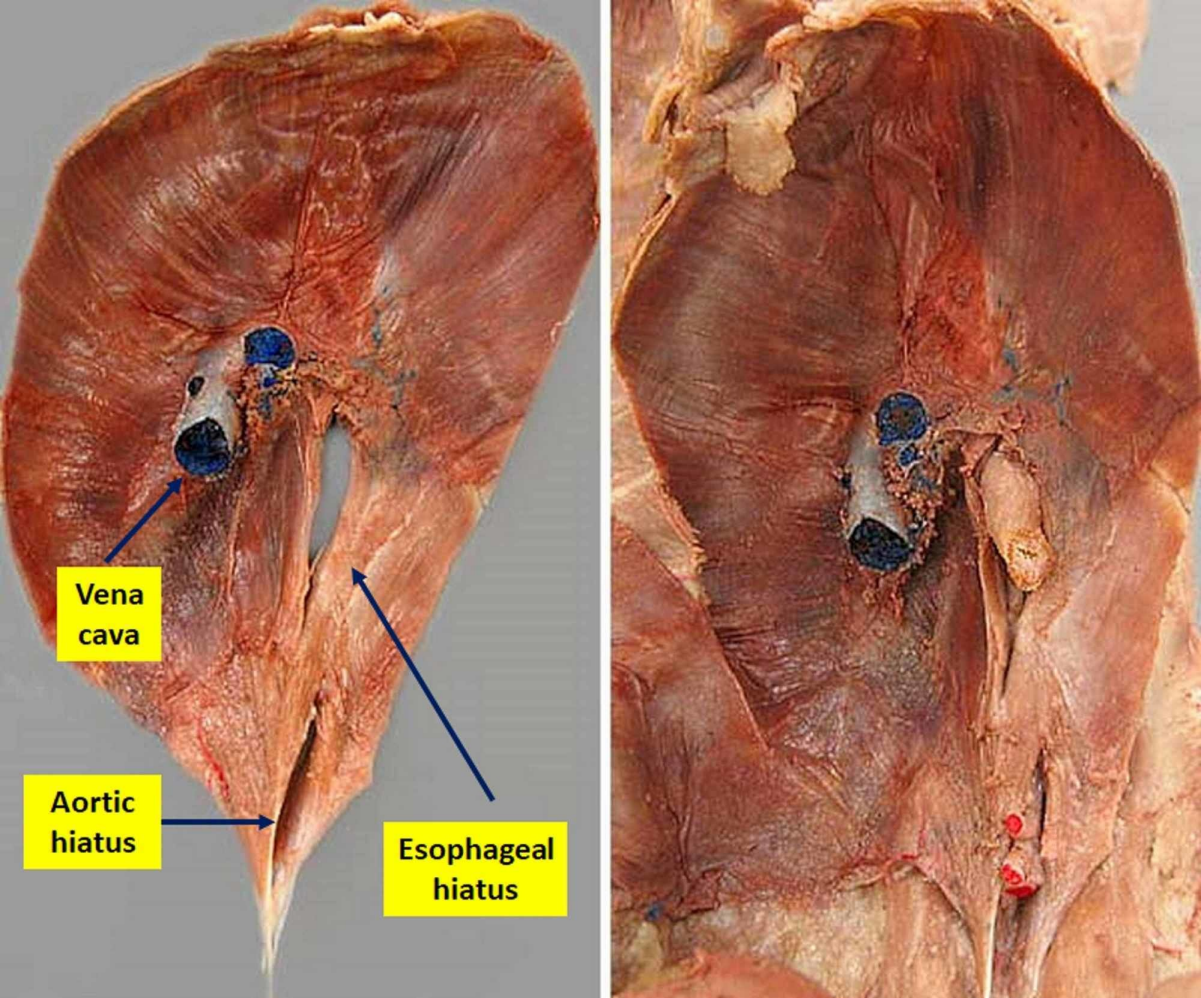
An anatomical dissection diaphragm from its abdominal position. On the left, the aorta and esophagus have been removed, while on the right it is possible to observe the esophagus

The phrenic nerve undergoes a lower paracrine production of brain-derived neurotrophic factor (BDNF) and a negative adaptation of peripheral synaptic exchanges; the number of phrenic neurons decreases as well as the dendritic volume (on animal model) [19,20]. The same happens at the muscular level of the diaphragm. There is less neuregulin type 1 and 2 (NRG-1 and 2) with reduced protein synthesis (less muscle fiber repair) (Figure 2) [19].

A recent study of mice with an early ageing pattern found a diaphragmatic situation of hypertrophy, which would seem at odds with previous literature [21]. In fact, in the early stages of ageing, the ultimate process will lead to processes of atrophy, and we witness a remodeling of the muscle, trying to repair the weakening fibers and mimicking hypertrophy (pseudohypertrophy). The same mechanism is found in some initial pathological conditions such as Duchenne muscular dystrophy [22]. Another study on a human model and with ultrasound evaluation highlighted a thickening of the diaphragm muscle [23]. This adaptation is most likely due to a contraction of the diaphragm muscle, called into question in a more frequent way for the lumbar stabilization. The elderly person has a greater difficulty maintaining balance and has an altered posture of the column; the deep hamstrings (multifidus muscle) are weaker and thinner, resulting in a weaker control of the lumbar area [23]. The diaphragm plays an important role in keeping the lumbar tract stable and contracts regardless of the breathing [24]. The diaphragm changes its morphology, becomes flatter and less elastic (increases stiffness), with a lower shortening speed [18,25].

Vagus nerve and intercostal nerves in ageing

The vagus nerve innervates the portion of the esophageal breath, while the intercostal nerves innervate the paracostal and intercostal muscles, which allows better movement of the coasts during the act of breathing [26,27]. The adaptations that the vagus nerve undergoes in the presence of ageing are likely to be related to the thicker (somatomotor) vagal fibers, which thin out [28]. Further research is needed to obtain more information on the mechanistic pathways that result in vagal nerve degeneration. About intercostal nerves, there is a lack of concrete data on the functional and morphological adaptation of nerve tissue. We can indirectly say that they are activated (animal model) during breathing to a greater extent when compared to young subjects, as there is a higher blood flow call [29]. Electromyography studies lack a higher blood flow call [29]. They are also lacking in uniqueness and this renders it difficult to obtain objective data [30].

Diaphragm afferences and supraspinal relationships

The diaphragm plays an extraordinary role in the movement, muscle coordination, and posture. The phrenic nerve has high diameter (Ia, Ib, II) fibers, afferent fibers with a smaller diameter of non-myelinated type IV (C fibers), and type III myelinated free nerve endings [31]. The first type of afference is activated during the contraction of the diaphragm (inspiration), while the thinnest afferent type is activated during the inspiratory and exhalatory phase [31]. In particular, Ia-type fibers are activated during contractile fatigue, while Ib-type fibers are silenced during muscle fatigue of the diaphragm [31]. Probably, these two different afferents respond to mechanical stimuli or mechanoreceptors and focal pressure stimuli (similar to Pacini receptors) [31]. The afferences with smaller diameters come in greater numbers and are classified according to their properties: ergotropic and metabotropic. Diaphragm fatigue activates type IV terminations but not those of type III [31]. Myelinated afference with a large diameter also carries visceral information. The afferences that come to foil VII, VIII, and IX have synaptic connections (interneurons) with the brake motor neurons, whose functions are still unknown to us, and with motor neurons involving the ancillary respiratory muscles [31]. In the spinal cord, the activation of the brake nerve on one side evokes the counter-side response (phrenic reflex) of the opposite phrenic nerve [31]. The phrenic afferents reach the cerebellum, the limbic area (thalamus, amygdala, pre-frontal cortex, periaqueductal grey area, hypothalamus, pituitary), and the somatosensory cortex via spino-bulbar pathways and spinothalamic pathways [31]. The cortex possesses bilaterally the positional representation of the diaphragm through the phrenic and not as a purely spinal metameric element. Furthermore, both sides of the cortex where the position of the diaphragm muscle is represented can communicate via the corpus callosum (cortico-cortical pathway) [31].

Vagal diaphragm afference and supraspinal relationships

**Figure 3:**
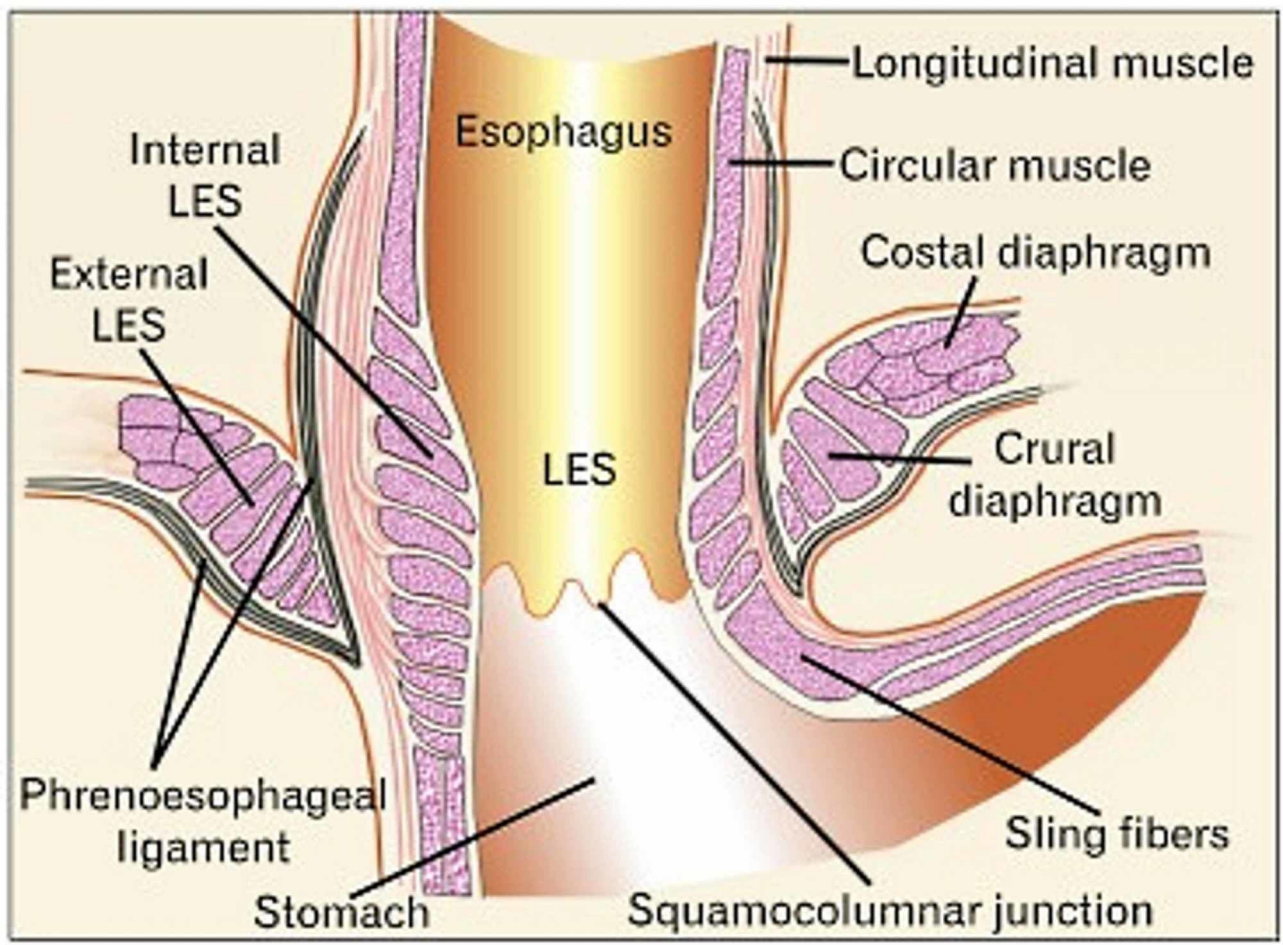
The area of passage of the esophagus inside the diaphragm and the phrenoesophageal ligaments

Diaphragmatic vagal afferents come from the diaphragmatic crura and the phrenoesophageal ligament (or the membrane of Laimer), in particular, from mechanoreceptors during transient lower esophageal sphincter relaxations (TLESR) (Figure 3) [32].

There are receptors that have a high response threshold (activated for strong mechanical stimuli) and low-threshold receptors (less than 10 mg of the applied voltage); these mechanoreceptors are not activated for stretching stimuli and are silent at rest [32]. The relevant information of the vagal receptors reaches the medulla oblongata and the Ganglion nodosum to finally reach the nucleus of the solitary trait. They are sensitive and visceral afferents that make up the vagal information from the diaphragm [32]. The vagal diaphragmatic information reaches the nucleus of the solitary trait and will eventually be brought to the vestibular and limbic area [33]. The vagal efferences of the esophageal breath, derived from the rostral area from the level of obex within the lateral regions of the nucleus of the dorsal motor, are of the cholinergic and excitatory type and are independent of cardiac and respiratory activity [32].

Alteration of force in the presence of diaphragmatic dysfunction

We know that the healthy elderly has a decline in expressed strength and a decrease in limb muscle coordination, resulting in a decrease in diaphragmatic force [11,18]. A healthy diaphragm muscle is able to stimulate baroceptors (adventure of the aortic arch and carotid glomus), particularly during a deep inhalation, which reach the nucleus of the solitary tract [34]. The nucleus of the solitary trait will send an efferent inhibitory response to the medullary ventrolateral rostral area of the pre-sympathetic area, and muscle strength and coordination improve [35]. The breathing bilaterally activates the primary cortex (M1), cortex premotor, and neighboring motor areas; these brain areas improve the response of muscle strength and performance, even by an extra 10% [36]. Breath from the nose (deep and rhythmic) produces rhythmical oscillations that propagate in different areas of the brain, including the limbic area (emotions) and hippocampus (memory) and, probably, oscillations emphasized by the pyriform cortex and olfactory bulb [36]. These oscillations, especially gamma waves (40-150 Hz), allow the neural network to function better, temporally improving the synaptic contact duration and the same electrical activity and stimulating synaptogenesis [36]. The same diaphragm muscle with phrenic afference stimulates delta and theta (low frequency) brain waves, stimulating areas such as the insular cortex and somatosensory (gesture storage) [36]. The diaphragm muscle plays a key role in lumbar stability aids the proper motor function of the limbs, as during a walk, thanks to the abdominal pressures (Pdi) that are created [37]. The elderly people have characteristics that can link to a diaphragmatic dysfunction. The strength of the diaphragm has decreased, as has the duration of prolonged stress (cough) [18]. In light of available data, we can hypothesize a negative influence of the adaptation of the diaphragm muscle to the decline of certain functions, such as the motor incoordination of the limbs and a higher percentage of falls, as well as cognitive decline. The percentage of low-back pain feedback in healthy older people is high, and one of the causes is a dysfunction of the diaphragm muscle, which does not adequately support the movement of the lumbar vertebrae [24,38,39]. In older people, baroreceptor afferents are altered, with higher sympathetic system activity values [40]. An altered baroreceptor function leads to vestibule dysfunction and consequent motor control dysfunction and dizziness [41]. This altered condition leads to uncertainty in the path, a proprioceptive muscle alteration, and increased fatigue [14]. A rehabilitative approach to the recovery of the diaphragm muscle leads to motor, local, and systemic improvements [23,42]. Dizziness warns cause fear of moving and walking to avoid accidental falls, further reducing individual independence [14]. The speed of the pitch decreases, negatively affecting the survival rate over the long period [14]. The diaphragm muscle in a condition of fatigue (cough for prolonged periods) activates the phrenic afferents (III and IV), which will stimulate the activity of the sympathetic system to the skeletal muscles with greater vasoconstriction, in a closed circle of fatigue and motor and respiratory incoordination [43].

Emotional changes in the elderly and diaphragmatic dysfunction

**Figure 4:**
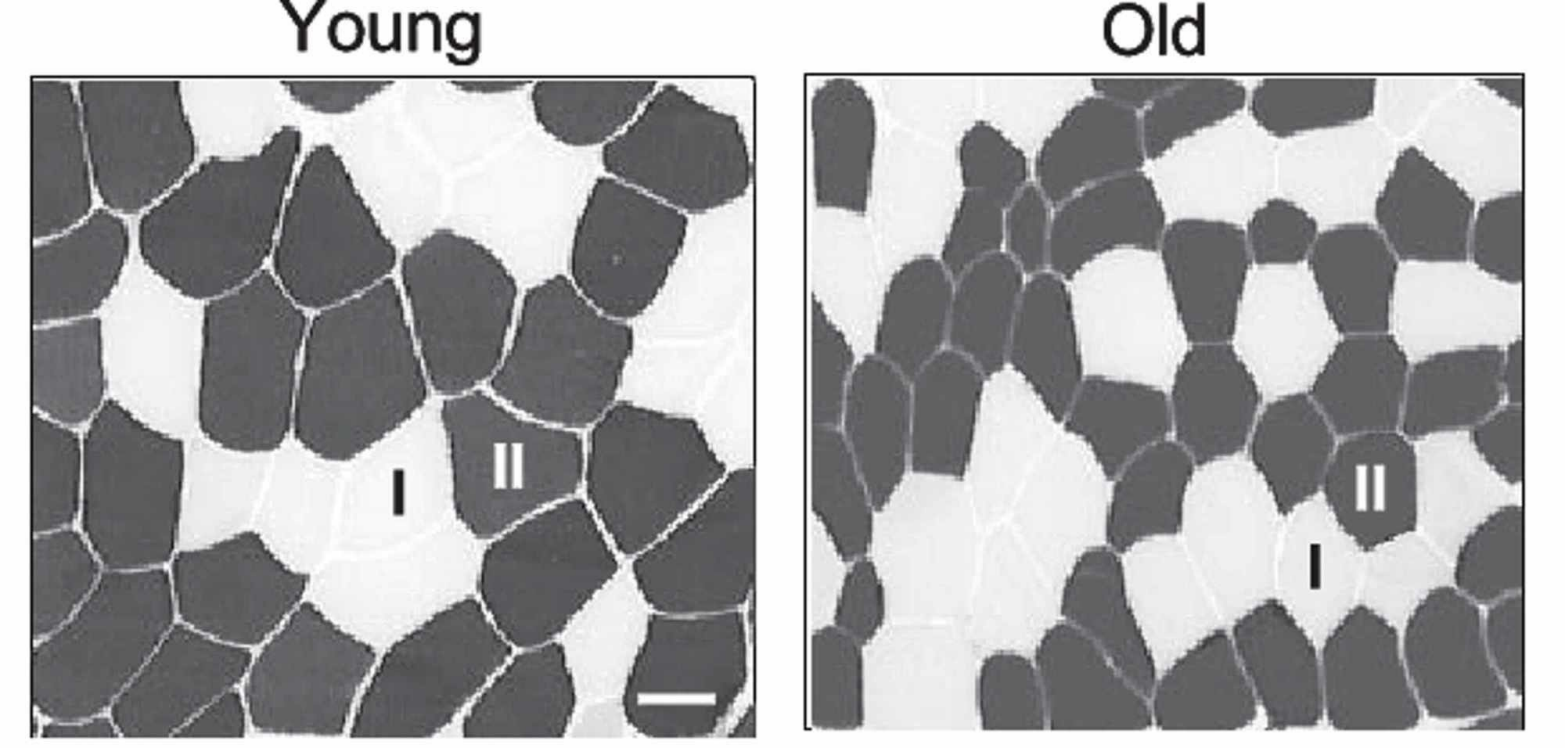
Biopsy of mouse muscle fibers Young mouse (left); old mouse (right). The aerobic fibers in dark shade are I, while the white or anaerobic fibers are II (as labeled)

Decreased motor independence leads to depressive conditions, especially in women [14]. Depression, anxiety, and fear of falling can also correlate to diaphragm muscle dysfunction. An altered position and morphology of the diaphragm with decreased protein mass, as in the adaptation of the elderly, reflects other systemic pathologies, where concurrent emotional and respiratory alterations are found (Figure 4) [33,44].

A diaphragmatic muscle with such adaptations moves with a smaller range and is less elastic; all nerve structures that pierce the muscle will undergo negative adaptations, like any other somatic or visceral nerve that undergoes a stretching, trapping, or a chronic crush. Healthy elderly people have a higher systemic level of inflammatory substances (inflammageing), compared to healthy non-elderly subjects [45,46]. When the diaphragm is fatigued, it produces inflammatory substances [47]. The reduction in blood volume to the diaphragm muscle could alter the nerve function. Dysfunction of the vascular endothelium due to increased levels of endothelin-1 and reactive oxygen species and a decrease in nitric oxide availability stimulate cascading a non-physiological reaction of the sympathetic system [48]. Less blood perfusion in proximity to the brake or vagal drive plates could further alter their function [49]. By combining all this information, we can strongly assume that the brake and vagal afferents from the diaphragm muscle, and those that arrive in limbic areas, will not properly solicit emotional areas in a physiological way, leading to potential mood changes.

## Conclusions

We aimed to discuss adaptations of the diaphragm muscle in the presence of ageing and some other ailments and co-morbidities. The decline of MIP MEP correlates with an increase in mortality and morbidity. We also sought to relate back pain, emotional alterations, motor incoordination, and cognitive disorders with functional alteration of the diaphragm muscle. Even though we have an enormous amount of data to support these consequential reports, there is a lack of more in-depth data to reach conclusions with an absolute degree of certainty. We hope this article would encourage researchers and clinicians to further investigate these not-so-comprehensive and un-elucidated issues.
